# Selective Solvolysis of Bio-Based PU-Coated Fabric

**DOI:** 10.3390/polym14245452

**Published:** 2022-12-13

**Authors:** David De Smet, Jente Verjans, Myriam Vanneste

**Affiliations:** Centexbel, Technologiepark 70, 9052 Zwijnaarde, Belgium

**Keywords:** solvolysis, polyurethane (PU), coating, fabric, bio-based

## Abstract

Polyurethane (PU) coatings are widely applied on high performing textiles due to their excellent durability and mechanical properties. PUs based on renewable resources were developed to improve the environmental impact of coatings by decreasing the carbon footprint. However, at the end-of-life, PU-coated textiles still end up as landfill or are incinerated since PUs are not biodegradable and are not being recycled at this moment. Therefore, the recycling of PU-coated substrates needs to be examined. This study reports the selective solvolysis of a polyester (PET) fabric coated with a bio-based PU using a 70% ZnCl_2_ aqueous solution. This method allowed the easy separation of the coating from the fabric. The thermal, chemical and mechanical characteristics of the virgin PET and recycled PET were examined via tensile strength tests, IR, TGA, DSC and GPC. Analysis of the fractions after solvolysis revealed that the PU was converted into the original polyol and an amine, corresponding to the isocyanate used for PU synthesis.

## 1. Introduction

Due to its versatility and durability, polyurethane (PU) is widely used in diverse applications such as coatings, foams, adhesives, sealants and elastomers. For properties such as abrasion resistance, flexibility, durability to washing, PU outperforms other polymers that are frequently used in coatings, which makes them suitable for durable textile applications. Two-component (2K) PU coatings are a promising alternative for current solvent based (e.g., N,N-dimethylformamide (DMF)) coatings to comply with all regulations. Indeed, DMF is a substance of very high concern (SVHC) and might be prohibited in the EU [1].

Next to the environmental and health concerns related to solvent-based PU coatings, another aspect to take into account is the end-of-life pathways of PU materials. Increasing production and application of PU in products that wear out over time (e.g., clothes, shoes), or that are exchanged for other ones (e.g., cars, furniture), result in an accumulation of post-consumer waste. Due to the low susceptibility of PU to physical, chemical and biological factors, and the toxicity of some of the combustion products, landfilling is still the most common way to process PU waste and PU-coated textiles. Efforts need to be made to overcome landfilling of PU-coated textiles, but this often implies separation of the PU coating and textile before it can be further processed. Possible solutions for end-of-life polymers are debonding on demand, by applying an external stimulus such as light and temperature, the use of degradable polymers, or solvolysis. In the case of stimuli-responsive polymers, light is often used as a possible trigger to induce structural changes [2,3,4]. Photoresponsive delamination can be achieved by combining pH-responsive polyacrylate copolymers with a light sensitive additive. The light sensitive additive could be a photoacid, which decomposes upon UV irradiation [5,6,7]. Akiyama et al. reported a series of azobenzene compounds which can photoisomerize between the cis and trans configuration when exposed to a suitable wavelength, resulting in reversible bonding and debonding [8]. Another method that uses light as a trigger for debonding is the incorporation of photocleavable groups within the adhesive polymer networks. A photoreactive o-nitrobenzyl segment could be incorporated into the backbone of an adhesive polyester to generate a switchable bonding system [9]. Li et al. reported a photocleavable adhesive polymer bearing o-nitrobenzyl ester linkage along its backbone. UV irradiation induced the degradation of the polymer backbone and decreased the adhesion strength [10]. 

Because the development of light-responsive debonding-on-demand PU coatings requires the synthesis and incorporation of photoreactive segments in the PU backbone, the mechanical properties and durability of these PU materials are often inferior compared to conventional PUs. Therefore, next to debonding-on-demand, another option for delamination is to selectively degrade the PU coating via solvolysis, i.e., the reaction with a solvent at elevated temperature and/or pressure, which can be catalyzed by an acid or a base [11]. Sanchez-Cadena and coworkers investigated the solvolysis for thermosetting polyacrylate-urethane coatings using diethylene glycol catalyzed by KOH [12]. Very recently, Miguel-Fernandez et al. reported the chemical recycling of rigid PU foam waste by catalytic glycolysis. After purification of the glycolysis reaction products, the polyols could be recovered. Subsequently, partial substitution of the commercial polyol by the recycled polyols for PU foam synthesis was investigated. Increased reaction rates were observed, which were possibly caused by the presence of aluminum oxide that was still present in the recycled polyols [13]. Pazdur and coworkers reported the multistage chemical recycling of PU (glycolysis with ethylene glycol followed by hydrolysis), resulting in an additional recovery of the amines originating from the isocyanate parts next to the polyols [14]. The glycerolysis of PU foam was also studied. The produced glycerolysate was used to partially replace commercial polyol in the prepolymer mixture. The polyol replacement with 25% did not cause significant changes in comparison with the reference PU [15]. In another study, rigid PU foams were subjected to a single-phase glycolysis process, which resulted in a pure recovered polyol (71 wt% of polyol) [16]. Next to glycerolysis, rigid polyurethane foam was also depolymerized using diethylenetriamine and sodium hydroxide. At the end, the diamine originating from the diisocyanate (i.e., 4,4′-methylenedianiline) was recovered [17]. Another promising method for the recycling of PU foam waste streams is acidolysis. Grdadolnik et al. reported the use of adipic acid to cleave the urethane bonds combined with microwave heating, which significantly reduced reaction times. The purified recycled polyols were used for the synthesis of flexible PU foams [18]. PU foam waste could also be transferred to new nanocomposites, which can be applied as a low-cost antibacterial material and for removing synthetic dyes from industrial effluents [19]. Watondo et al. reported the degradation of flexible PU foam by an extruder with diethanolamine. The decomposed product could be used to replace virgin polyol in PU elastomers [20]. Alternatively, thermoplastic PU (TPU) elastomers were depolymerized in sub- and supercritical methanol. The resulting products consisted of the monomers of TPU and their methylates [21]. Another method to disintegrate PU elastomer scraps is by glycerolysis using distilled and waste (crude) glycerine. Glycerolysates containing a relevant percentage of recovered polyester polyol were obtained. The recovered glycerolysates contained carbamates and amines [22]. Recently, Li et al. reported the synthesis and chemical recycling of TPU elastomers from bio-based δ-caprolactone. By simple thermolysis of the synthesized polymers, the pure δ-caprolactone monomers could be recovered [23]. Lastly, Hou and coworkers reported the selective degradation of PU elastomers by cleavage of C–O and C–N bonds in 70% ZnCl_2_ aqueous solution. This catalyst-assisted solvolysis did not degrade the polyether bonds and resulted in the amine form of the used isocyanate (2,4-diaminotoluene) and the original polyether polyol (polytetramethylene ether glycol) [24].

As described above, many efforts have been made to recycle PU foams or elastomers. Indeed, the weight fraction of PU in these products is higher compared to the weight fraction of PU in coated textiles, which makes it easier to obtain higher amounts of polyols, amines or other degraded products in high purity starting from foam or elastomers. Furthermore, in the case of coated textiles, the PU degradation process should not heavily affect the textile to enable the reuse of the recovered fabrics. However, selective chemical degradation of a PU binder does not necessarily require debonding of the coating, since it does not degrade the fabric and it allows facile recycling of the fabric afterwards. The degradation products of the coating can be used as building blocks for new chemicals. In this study, we report the selective degradation of a PU-coated textile by degrading the PU in polyol and amine, without prior need for delamination of the coating from the fabric. The structural, mechanical and chemical characteristics of the virgin polyester and recycled polyester were found to be similar. The solvolysis of the PU coatings was carried out in an aqueous ZnCl_2_ solution at a concentration of 70% ZnCl_2_. Comparable to deep eutectic solvents (DES) and ionic liquids (IL), the aqueous ZnCl_2_ solution showed Lewis acid properties and efficient catalytic activity, but at a lower price point, which makes the ZnCl_2_ method more economically attractive.

## 2. Materials and Methods

### 2.1. Materials

Bismuth neodecanoate was purchased from Sigma-Aldrich (Diegem, Belgium). Desmodur eco N 7300 was kindly provided by Covestro (Leverkusen, Germany). Zinc chloride (ZnCl_2_), sodium chloride (NaCl), diethyl ether and ethyl acetate were bought from ChemLab (Zedelgem, Belgium). Priplast 3172 was kindly provided by Croda (Gouda, The Netherlands). Woven polyester fabric (105 g/m^2^) was purchased from Concordia Textiles (Waregem, Belgium). The brine solution (saturated NaCl in water) was prepared by adding an excess of NaCl to distilled water.

### 2.2. Synthesis of PU Coatings

The PU coatings were prepared by reacting Priplast 3172 (OH value: 41 mg KOH/g) with Desmodur eco N 7300 (NCO content: 21.9%) in molar equivalent ratio 1:1 (NCO:OH) using bismuth neodecanoate as a catalyst. Priplast 3172 (63 g) was mixed with bismuth neodecanoate (0.05 g). Subsequently Desmodur eco N 7300 (8.88 g) was added to the polyol mixture. The 2K formulation was applied to a polyester fabric via knife over roll with a coating thickness of 100 µm, and the coating formulation was cured for 2 minutes at 155 °C. 

### 2.3. Degradation of PU-Coated Textile

**Figure 1:**
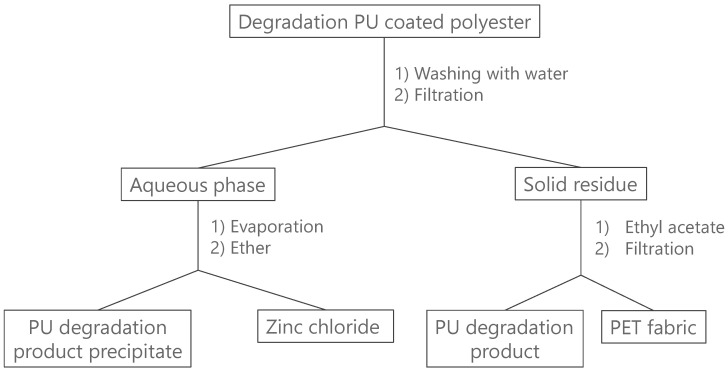
Flow chart of the degradation of PU-coated polyester and purification.

PU-coated polyester (10 g) was added to an aqueous ZnCl_2_ solution (70 wt% in water, 250 g). The solution was heated to 140 °C and kept at this temperature for 2 h. After the reaction, the degradation products were cooled to room temperature and stirred into 250 mL water for 5 min. The mixture was filtrated resulting in an insoluble residue (fabric and insoluble degradation products of PU) and a water mixture. The residue was poured into 200 mL ethyl acetate and the fabric was filtered off and subsequently dried overnight at 70 °C under atmospheric pressure. The ethyl acetate mixture was washed three times with brine (saturated aqueous NaCl solution) before evaporating the organic phase with a rotary evaporator. The water mixture was evaporated and diethyl ether was added. The precipitated product was separated via filtration and dried overnight at 70 °C under atmospheric pressure. A flow chart of the procedure is shown in Figure 1.

### 2.4. Characterization

Infrared spectra (IR) (in µ-ATR mode) were recorded using a Nicolet 6700 spectrometer from Thermofisher Scientific (Waltham, MA, USA). A spectral range from 500 to 4000 cm^−1^ with a resolution of 4 cm^−1^ was used. The infrared analysis was used to characterize the 2K PU, polyester textile and degradation products after recycling. The surface morphology of the polyester fabric was visualized using field emission gun scanning electron microscopy (FEG-SEM) (JSM 7600 F from Jeol Europe (Zaventem, Belgium)). To prevent charging, the specimens were sputtered with a palladium coating.

Thermogravimetric analysis (TGA) was used to investigate the thermal decomposition using a Q500 thermogravimetric analyzer (TA Instruments (Asse, Belgium)). Samples were conditioned at 23 °C and 50% relative humidity. Analyses were performed in air with a ramp rate of 10 °C/min from 30 °C to 600 °C. Differential scanning calorimetry (DSC) analysis of polyester was performed to measure the glass transition temperature (T_g_) and melting point (T_m_) using TA Instruments Discovery DSC2500 from TA Instruments (Asse, Belgium). All samples were conditioned at 23 °C and 50% relative humidity and heated from 0 to 300 °C, cooled down from 300 to 0 °C and heated back from 0 to 300 °C during analysis. Analyses were performed with a heating and cooling rate of 10 °C/min. The crystallinity (X_c_) of virgin and recycled polyester was defined according to the equation:

X_c_ (%) = (ΔH_m_/ΔH_0m_) × 100% (1)

where ΔH_m_ is the melting enthalpy of PU and ΔH_0m_ is the melting enthalpy of 100% crystalline PET (140 J/g) [25].

Elongation at break of fabrics was determined using Instron electronic fabric tension tester from Instron (Norwood, MA, USA) according to ISO 13934-1. The tension loading speed was 100 mm/min. 

The molecular weight of virgin and recycled polyester was determined via gel permeation chromatography (GPC) using a Waters 1515 equipped with Waters 717 autosampler and Waters 2998 PDA detector using a phenogel column. The elution solvent was hexafluoroisopropanol and poly(methyl methacrylate) standards were used to determine the molecular weights. Analysis was carried out at room temperature and the flow rate was set at 1 mL/min.

## 3. Results

### 3.1. Structural Characterization of Bio-Based PU Coating

**Figure 2:**
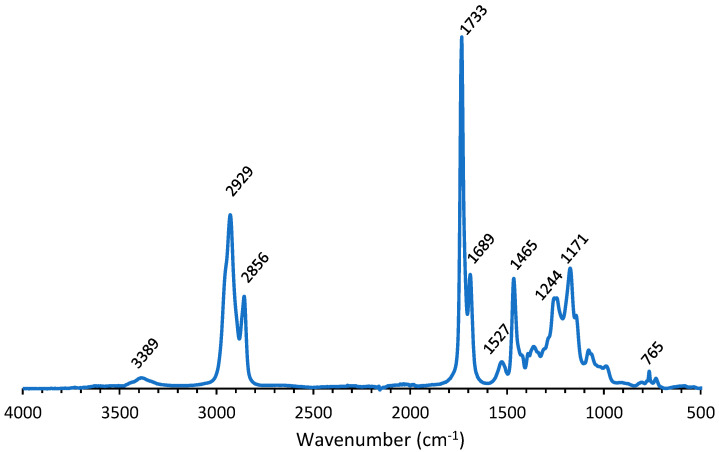
IR spectrum of bio-based PU coating.

The bio-based PU coating was characterized with IR. Figure 2 demonstrates the IR spectrum of the bio-based PU coating. Bands characteristic for PU appeared in the IR spectrum. Table 1 gives an overview of the different bands and the corresponding groups. The absence of OH and isocyanate groups and the appearance of NH bands in the IR spectrum is characteristic for completely cured PU coatings.

The band at 3389 cm^−1^ was attributed to NH stretching, while the peaks at 1733 and 1689 cm^−1^ corresponded to C=O stretching of the urethane and ester moieties (Table 1). The peak at 1527 cm^−1^, originating from N–H bending and C–N stretching, is characteristic for polyurethanes. The band at 1244 cm^−1^ was assigned to C–O–C urethane vibrations and the band at 765 cm^−1^ corresponded to COO urethane vibrations [26,27,28,29]. 

### 3.2. Characterization and Analysis of Degradation Products

**Figure 3:**
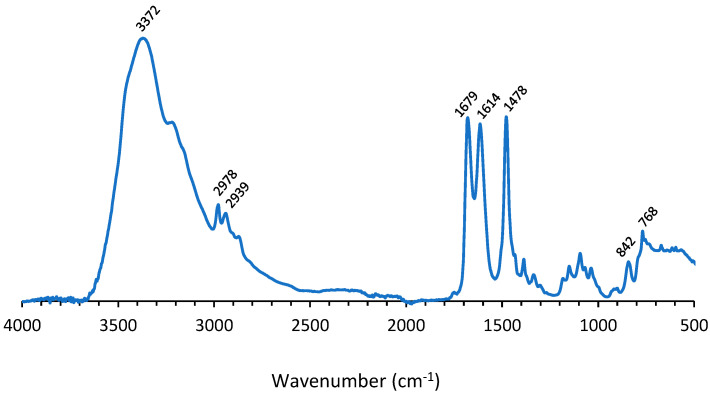
IR spectrum of degradation product extracted out of the water phase upon degradation of PU coating.

The degradation experiments were performed in an aqueous solution with 70 wt% ZnCl_2_ at 140 °C. Urethane bonds in PU can hydrolyze in water at elevated temperatures. Wang et al. reported that the urethane bonds and urea linkages were completely cleaved at 140 °C by the coordinated catalysis of unsaturated coordinate Zn^2+^ [24]. Upon heat treatment in 70 wt% ZnCl_2_, the aqueous phase was evaporated and diethyl ether was added. Figure 3 shows the IR spectrum of the degradation product which was insoluble in diethyl ether.

The absorption band at 3372 cm^−1^ corresponded to NH stretching. The peak at 1614 cm^−1^ was attributed to NH bending. This signal is only seen in the case of primary amines, which indicates that the degradation product is a primary amine. The band at 842 cm^−1^ corresponded to NH wag. The presence of isocyanurate was verified by the bands at 1679, 1478 and 768 cm^−1^, originating from C=O stretching vibration in the isocyanurate ring, CH_2_ asymmetric bending vibration when attached to isocyanurate and the skeletal stretching vibration of isocyanurate ring, respectively [30]. The peaks at 2978 cm^−1^ and 2939 cm^−1^ were attributed to −CH_2_ stretching, while other modes of −CH_2_ vibrations corresponded with the band at 1478 cm^−1^.

**Figure 4:**
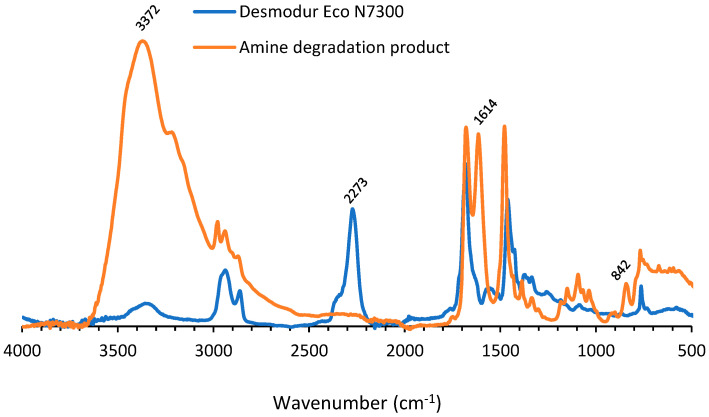
IR spectrum of amine degradation product and Desmodur eco N 7300.

The IR spectrum of the amine showed high similarity with the IR spectrum of Desmodur eco N 7300, indicating that during the ZnCl_2_ treatment, the amine derivative of Desmodur eco N 7300 was produced (Figure 4). The main differences between both spectra were the appearance of an isocyanate peak at 2273 cm^−1^ in the IR spectrum of Desmodur eco N 7300 and the absence of the bands for a primary amine at 3372 cm^−1^, 1614 cm^−1^ and 842 cm^−1^.

**Figure 5:**
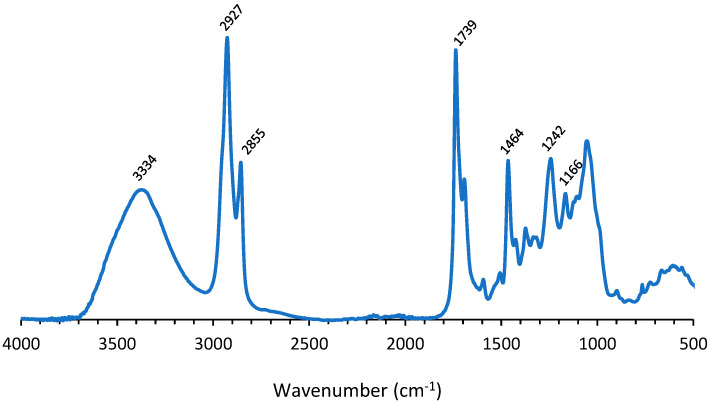
IR spectrum of degradation product insoluble in aqueous phase (recycled polyol).

**Figure 6:**
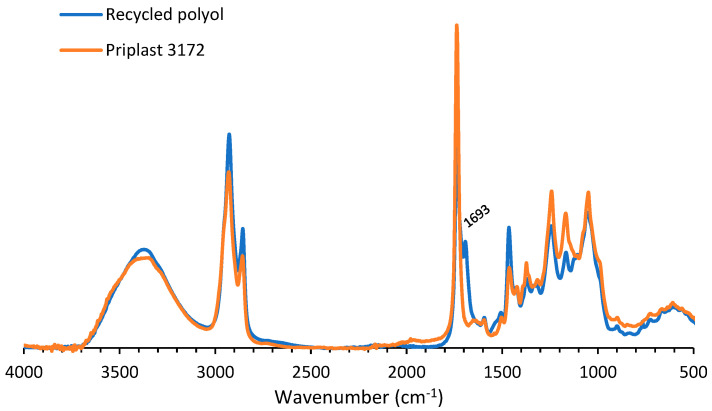
IR spectrum of recycled polyol and Priplast 3172.

Figure 5 exhibits the IR spectrum of the degradation product insoluble in the aqueous phase. The spectrum showed a band at 3334 cm^−1^ which could be attributed to OH-stretching. The band at 1739 cm^−1^ could be assigned to C=O ester vibration, while the peaks at 1240 and 1166 cm^−1^ originated from C–O vibration in esters and ethers. The bands at 2927 cm^−1^ and 2855 cm^−1^ corresponded with −CH_2_ stretching, while other modes of −CH_2_ vibrations resulted in the band at 1478 cm^−1^. Since the results seem to indicate that a polyester polyol was found in the non-aqueous phase after treatment of the coated fabric with ZnCl_2_, a comparison was made between the IR spectra of the found polyol and Priplast 3172 (Figure 6). The IR spectra corresponded very well, except for the band at 1693 cm^−1^, corresponding to the C=O stretch of aldehyde group. This might indicate that during treatment with ZnCl_2_, a part of the OH groups of the polyol were oxidized to aldehyde groups.

### 3.3. Characterization of Recycled Polyester

**Figure 7:**
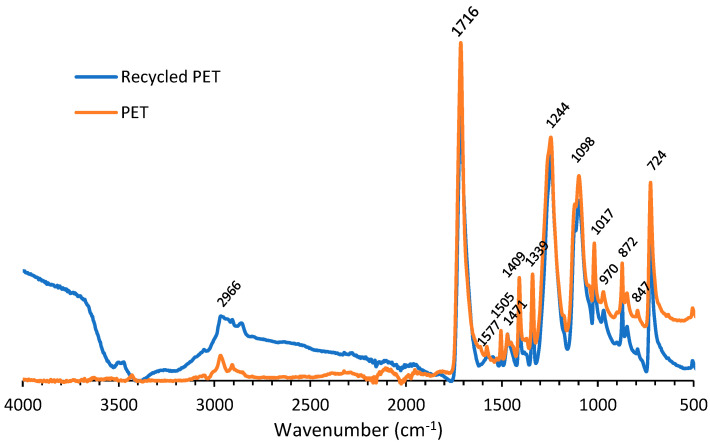
IR spectra of recycled and virgin PET.

Figure 7 demonstrates the IR spectra of virgin polyester and recycled polyester. Both spectra showed significant similarities, indicating that the PET recovered upon the degradation of PU in ZnCl_2_ solution showed no degradation. The band at 2966 cm^−1^ could be attributed to C–H stretching, while the peak at 1716 cm^−1^ originated from C=O stretching. The signals at 1577 and 1505 cm^−1^ were allocated to aromatic C=C stretching. The peaks at 1471, 1409 and 1339 cm^−1^ corresponded to C–O stretching of the OH group and bending and wagging of the ethylene glycol segment, respectively. The peak corresponding to C–O stretching of the ester group appeared at 1244 cm^−1^. Peaks at 1098 and 1017 cm^−1^ could be attributed to the methylene group and vibrations of the ester C–O bond. Bands at 970, 872 and 847 cm^−1^ originated from the 1,2,4,5-substituted aromatic moieties. The signal at 724 cm^−1^ was ascribed to the interaction of ester groups with benzene rings [31,32].

**Figure 8:**
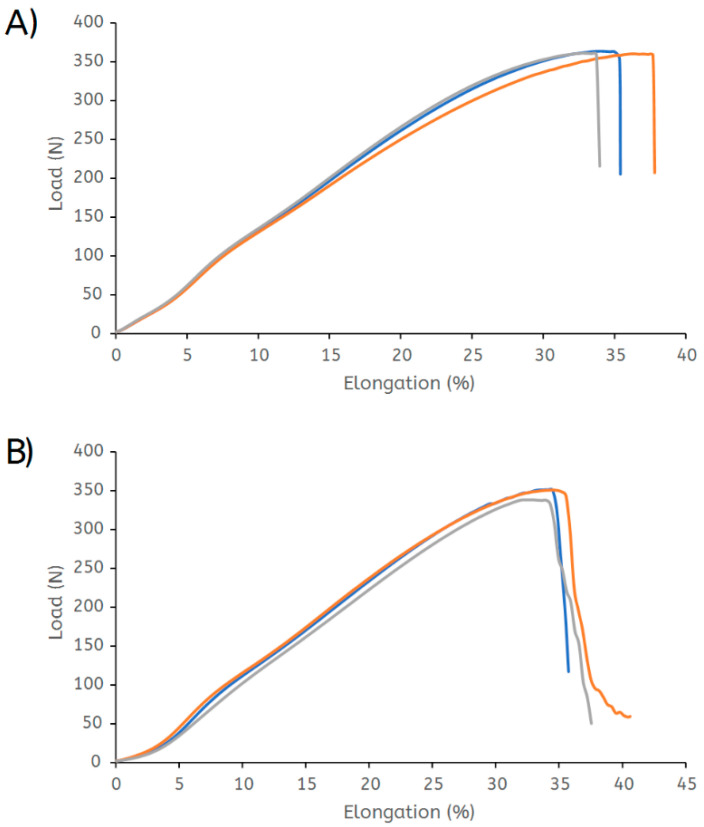
Tensile curves of (**A**) virgin and (**B**) recycled polyester.

The tensile force is defined as the maximum recorded force or load when a test specimen is ruptured during a test under the specified conditions (ISO 13934−1). The tensile force and elongation of virgin polyester fabric amounted to 360 N and 33.5%, respectively (Figure 8). Recycled polyester fabric had an elongation of 34% and tensile strength of 350 N. Thus, the thermal treatment in the presence of ZnCl_2_ did not affect the mechanical properties of the polyester fabric.

**Figure 9:**
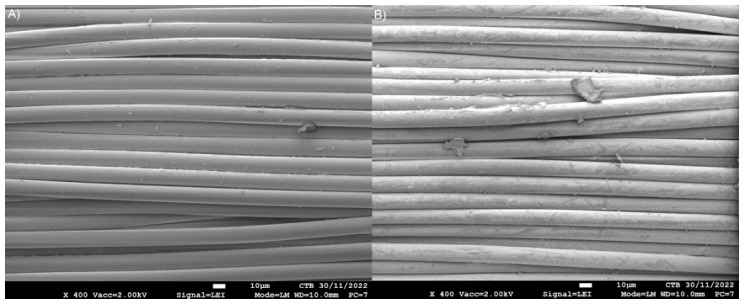
SEM analysis of (**A**) virgin and (**B**) recycled polyester fabric.

The surface morphology of both virgin and recycled polyester was analyzed via SEM (Figure 9). The analysis showed that the recycled polyester fibers were not degraded after the thermal treatment. However, a residue was observed on the surface of the recycled polyester fibers, which was not observed on the virgin material. EDX-analysis revealed that ZnCl_2_ was present on the recycled fabric, but not on the virgin fabric.

The molecular weight of both virgin and recycled polyester was examined via GPC. The number average molecular weight, weight average molecular weight and dispersity (Mn, Mw and Ð, respectively) were assessed and are displayed in Table 2. The dispersity is defined as the ratio Mw/Mn. The recycled PET showed no decrease in molecular weight compared to virgin PET, which indicated that the recycled PET did not degrade during solvolysis. This result is consistent with the data of the IR analysis.

### 3.4. Thermal Degradation of Polyester

**Figure 10:**
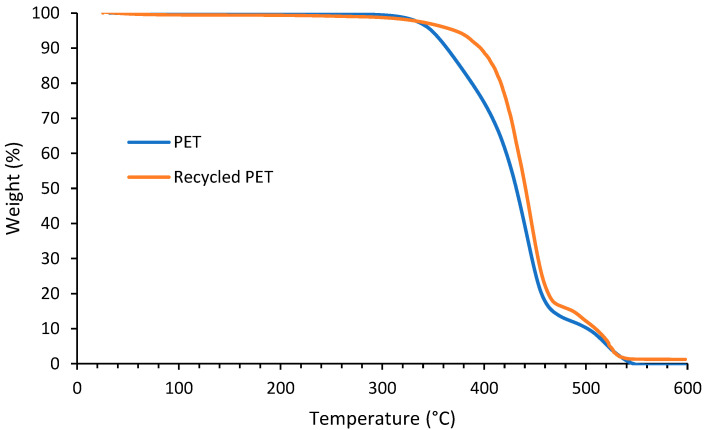
TGA curves of recycled PET and virgin PET.

Both virgin and recycled PET showed similar two-step thermal degradation (Figure 10). A significant mass loss occurred between 300 and 460 °C. The first degradation step involves the breaking of ester links. The thermal degradation is a result of intramolecular backbiting and hydrogen transfer resulting in cyclic oligomers. Several degradation products are formed in the first step including vinyl benzoate, terephthalic acid, benzoic acid and carbon dioxide. The second degradation step involves further degradation of the aromatic cyclic compounds [33,34]. Virgin PET tended to degrade at a lower temperature compared to recycled PET, since the degradation onset temperature, determined at 5% of mass loss, shifted from 348 to 371 °C. Further on, recycled PET showed a higher char residue at 600 °C. This could be attributed to the presence of residual ZnCl_2_ in the recycled PET.

### 3.5. DSC Analysis of Polyester

**Figure 11:**
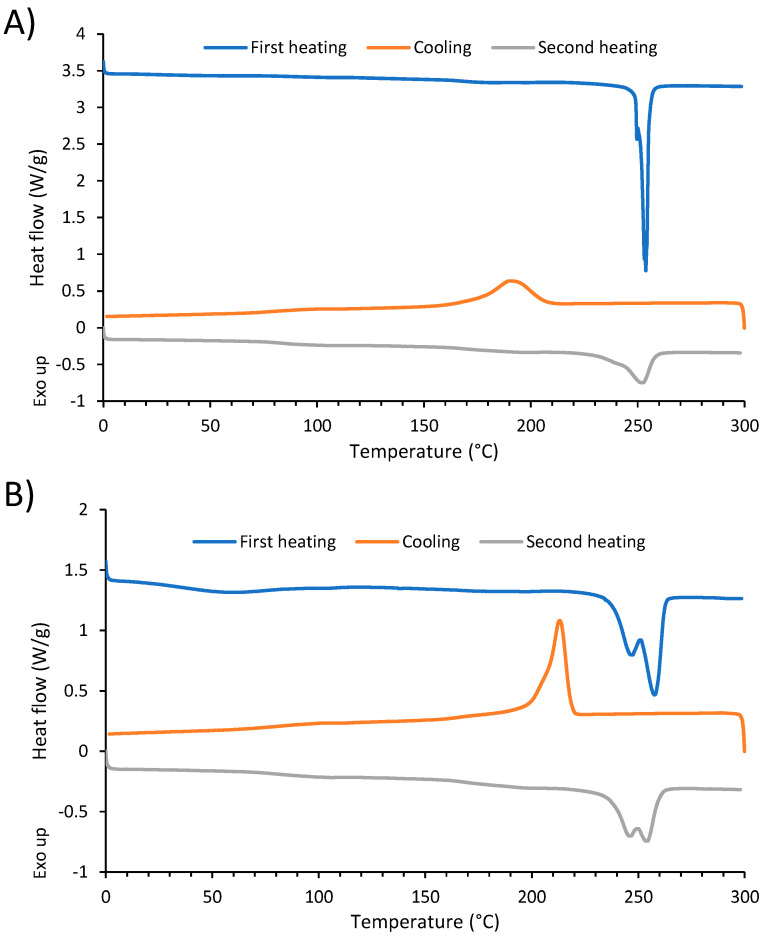
DSC curves of (**A**) virgin PET and (**B**) recycled PET.

Table 3 demonstrates the crystallinity degree (X_c_), glass transition temperature (T_g_) and melting temperature (T_m_) of virgin and recycled PET. X_c_ is calculated from the second heating. The virgin and recycled PET showed similar T_g_ and T_m_ (Figure 11). The broad melting peak, which corresponds to the wide distribution of lamellae thickness, is caused by a wider molecular weight distribution [35]. During the second heating, recycled PET showed two endotherm melting peaks, which is due to the presence of a dual lamella thickness distribution generated during crystallization [36]. The dual lamella thickness was probably caused by the presence of ZnCl_2_ impurities. The crystallinity degree of recycled polyester was a bit higher compared to virgin PET. The thermal treatment of polyester with ZnCl_2_ increased the crystallinity, due to increased ordering of the polymer chains. However, the increased crystallinity had limited impact on the mechanical properties (recycled polyester had similar elongation and a slightly lower tensile strength compared to virgin polyester). 

## 4. Conclusions

Bio-based 2K PU was coated on polyester fabrics to examine the recyclability of PU-coated fabrics via solvolysis. The coated fabric was put in a 70% aqueous solution of ZnCl_2_ and heated to 140 °C for 2 hours. Afterwards the fabric was washed with ethyl acetate to remove the impurities resulting from the degradation of PU. The polyester was characterized via IR, TGA, DSC and GPC. The liquid fractions obtained after solvolysis were analyzed using IR. It was found that the polyester did not degrade during solvolysis, while the PU was converted into the original polyol and the amine corresponding to the isocyanate building blocks. In a future study, the conversion of the amine into isocyanate and the synthesis of PU out of the recycled polyol and isocyanate need to be examined and analyzed as well as the processing of the recycled PET into new fabrics.

## Figures and Tables

**Table 1:** Overview of functional groups in bio PU detected by IR.

PU	
Wavenumber (cm^−1^)	Corresponding Group
765	COO urethane (deformation vibration)
1171	C–O–C ester (elongation vibration)
1244	C–O–C urethane (elongation vibration)
1465	CH (deformation vibration)
1527	N–H and C–N amide
1689	C=O urethane (elongation vibration)
1733	C=O ester (elongation vibration)
2856	CH (elongation vibration)
2929	CH (elongation vibration)
3389	NH (elongation vibration)

**Table 2:** Molecular weight (M_n_ and M_w_) and dispersity (Ð) of virgin and recycled polyester.

	M_n_ (kg/mol)	M_w_ (kg/mol)	Ð
Virgin PET	4.3	15.2	3.33
Recycled PET	4.9	18.2	3.68

**Table 3:** T_g_, T_m_ and X_c_ of virgin and recycled PET.

	T_g_ (°C)	T_m_ (°C)	X_c_ (%)
Virgin PET	83	252	39
Recycled PET	80	253	45

## Data Availability

The data presented in this study are available on request from the corresponding author.
